# The Effects of Traditional Concepts on Personal Values Among University Students in China

**DOI:** 10.3389/fpsyg.2022.872768

**Published:** 2022-03-28

**Authors:** Jun Song, Gong Sun, Ruilin Cai

**Affiliations:** ^1^Department of Science and Technology Industry, Changshu Institute of Technology, Changshu, China; ^2^Business School, Changshu Institute of Technology, Changshu, China

**Keywords:** China, face, harmony, personal values, university students

## Abstract

It is widely agreed that China is experiencing a substantial transition toward modernization. However, both business behavior and day-to-day life in China are still greatly influenced and regulated by the traditional culture that has been embedded in Chinese society for thousands of years. Therefore, when studying social phenomena in China, researchers must take the indigenous cultural context into account. Focusing on the young generation in China, the authors explore the relationships between the most fundamental traditional concepts and various value dimensions. Three hundred and thirty-two university students in Southeast China took part in the survey and data were analyzed with SPSS. The result might help both scholars and practitioners to better understand the contemporary Chinese culture. The discussion and implications are also offered.

## Introduction

After more than 40 years’ reform and opening up to the West, China has become one of the two economic superpowers and the world’s largest FDI recipient. Nearly 1,000,000 foreign-invested firms have established operations in this ancient but vigorous country. It is predicted China’s GDP will surpass that of the United States and become the world’s largest around 2035. For global companies, understanding Chinese young generation’s values is fundamental to achieve future’s business success in this huge market and nowhere is this truer than in China.

Moreover, it has been evidenced that traditional culture can influence individuals’ values (Ralston et al., 1993, 1997). Although China has been experiencing a substantial transition toward modernization, social behaviors are still greatly affected and regulated by traditional culture (Yang, 1998). Therefore, when studying social phenomena in China, researchers must take the indigenous cultural context into account.

Chinese traditional culture consists of concepts originated from Confucianism, Taoism, Legalism, and Buddhism and has been evolving for thousands of years. Even for each element, taking for instance of Confucianism, it may vary in different eras because of the impacts of economic, political, and other social–cultural factors. Therefore, we believe it is not accurate to treat such a complex cultural system as an integrated variable. Instead, we select two most fundamental Chinese traditional concepts—face and harmony and investigate their impacts on the values endorsed by the new generation in China.

There is further implication of this study. The past 40 years’ globalization has introduced Western culture and lifestyles to China. This modernized or westernized values evolution in most emerging non-Western industrializing countries is defined as cultural convergence (Inglehart, 1990). One of the major arguments against cultural convergence is that traditionalism and modernity can co-exist (Smith and Bond, 1998; Leung et al., 2005). For example, empirical studies find that Chinese people in Singapore, Taiwan, and mainland China endorse both traditional and modern values (Yang et al., 1989; Chang et al., 2003; Zhang et al., 2003). Even as Western culture pervades, the influence of traditions remains strong (Wang and Lin, 2009; Fang, 2010). The seemingly conflicting and paradoxical forces reconcile in the society (Faure and Fang, 2008; Yang and Stening, 2013). Thus, there might be more complex relationships between traditional concepts and modernized values. The results may help social scientists from various disciplines to better understand the contemporary culture in this ancient country.

### Personal Values

The most prestigious values framework is Hofstede’s (1980) work that incorporates the dimensions of individualism–collectivism, power distance (PD), uncertainty avoidance, and masculinity. Individualism–collectivism depicts the relationship between the individual and the collectivity. PD reflects the tendency of accepting inequality. Uncertainty avoidance assesses the extent of tolerance for ambiguity and risk. Masculinity reflects the degree to which one stresses competition and achievement. The first three dimensions exert great influences on business behavior and were widely used in relevant studies (Kirkman et al., 2006; Soares et al., 2007; Taras et al., 2010). However, Hofstede’s framework has long been criticized for its conceptual problems (Brewer and Chen, 2007; Sun et al., 2014a). Especially the individualism–collectivism dimension, which is too broadly defined (Bond, 2002; Fiske, 2002). Therefore, it is necessary to identify more clearly conceptualized and precisely defined dimensions of values in the current research.

Firstly, Singelis et al. (1995) and Triandis and Gelfand (1998) used horizontal (emphasizing equality) and vertical (valuing hierarchy) distinctions to further differentiate individualism and collectivism. Horizontal individualists tend to express their uniqueness and independence but consider themselves equal to others in status; vertical individualists prefer to improve their status through competition. Horizontal collectivists lay stress on sociability and an egalitarian social system; vertical collectivists emphasize improving the status of one’s own social group in competition with out-groups (Maheswaran and Shavitt, 2000). Some scholars emphasize that the horizontal–vertical distinction is an important way to assess values in business research (Meyers-Levy, 2006; Shavitt et al., 2006). In addition, Sharma (2010) maintain that the dimension of uncertainty avoidance actually incorporates the sub-dimensions of risk aversion (RA) and ambiguity intolerance. RA defined as one’s unwillingness to take risk can substantially affect various organizational behavior in Asian context (Froese and Xiao, 2012; Froese, 2013). Hence, in this study we focus on horizontal–vertical individualism–collectivism, PD and RA among Chinese university students.

### Traditional Concepts

The strictly hierarchical social structure in China has existed for thousands of years. In such a society, everyone must observe the principle of propriety (*li*) and behave properly within the bounds of one’s position. It is believed that the ideal way to achieve an ordered society is through a well-defined hierarchy (King and Bond, 1985). People must accept this structure and defer to authority even when they do not want to. Self-control is an important quality in China, especially when one is less successful. This could explain why Chinese organizations usually adopt a paternalistic leadership pattern and a vertical organizational structure. Cheng (1990) maintains that social role, not the self, determines one’s behavior. Gao et al. (1996) found that in China the weight of one’s voice depends heavily on one’s social status. Thus they must give face to and maintain harmonious relationship with the powerful in order to gain benefit, which makes face and harmony become the most fundamental principle in China.

#### Face

Face refers to a sense of favorable social self-worth that a person wants others to have of him or her in a relational and network context (Goffman, 1967). It reflects one’s social self-esteem and desire to be respected during interpersonal interactions (Ting-Toomey and Kurogi, 1998). It is the core of prestige, status, and dignity. Although people in every society desire a favorable social image, it is a more obvious characteristic in Confucian society (Hu, 1944). In collectivistic societies people rely upon others and are very concerned about how they are perceived (Markus and Kitayama, 1991). Face is central to Chinese people and is a fundamental principle in social and business interactions in China and other Confucian countries (Redding, 1990). During social interactions people always try to gain and save face and avoid losing face (Hwang, 1987). Today the most efficient way of gaining face is by achieving distinctiveness and status through possessing brand name products. Thus, face is widely linked to materialism, status, and luxury consumption (e.g., Wong and Ahuvia, 1998; Liao and Wang, 2009; Li et al., 2015; Sun et al., 2017). Besides, people may also lose face when their behavior does not reach others’ expectation, which will lead to embarrassment. Hence face loss can stimulate consumers’ tendency toward impulse buying (Sun et al., 2021). Zhang et al. (2011) identify two correlated dimensions of face consciousness—desire to gain face and fear of losing face. Wang et al. (2019) find the two dimensions exert contrasting effects on fashion consumption. Hence, we treat desire to gain face and fear of losing face as separate dimensions in the present study.

#### Harmony

Confucianism emphasizes harmony with nature and with others, encouraging people to avoid confusion, competition, and conflict in order to achieve inner and interpersonal harmony (Kirkbride et al., 1991). Good relations with others are considered the source of happiness and social stability. Thus Chinese people restrain their emotions to create a non-aggressive, peaceful environment (Yau, 1988). Cocroft and Ting-Toomey (1994) find that the Chinese tend to use indirect and mild conflict styles to maintain smooth relationships, whereas United States respondents are more likely to use direct and threatening conflict styles with its emphasis on self-expression and personal achievement. However, although the Chinese seldom openly challenge others, genuine harmony which is holistic, sincere, and heartfelt is hard to establish (Leung et al., 2008). People preserve surface harmony by agreeing with others in public, but may still disagree in private. This strategic tolerance is employed while waiting for a better opportunity to attain one’s own goals (Leung et al., 2002). When surface harmony is not considered optimal, the Chinese may adopt direct confrontation (Leung and Wu, 1998). In these circumstances, harmony is more like an instrument used to pursue personal benefit. Leung et al. (2002) use disintegration avoidance to reflect this instrumental or superficial harmony. This type of harmony is especially prevalent in collectivist societies that are hierarchically structured and densely networked. Moreover, genuine harmony is termed as harmony enhancement that reflects an authentic affinity for harmony. Both of the two types of harmony are included in the current study.

## Materials and Methods

### Participants

The sample consisted of 332 undergraduate students at a university in Jiangsu province, Southeast China. The students were given a survey to complete in class. A cover letter assured them that their participation was voluntary and their responses would be confidential. Participants included 91 males and 241 females. The average age was 20.3 years.

### Measurements

Face was measured by 11 items that make up of two dimensions—*desire to gain face* and *fear of losing face* from Zhang et al. (2011). The scale identifies face as a general personality trait in the social context and achieves good convergent validity, discriminant validity, and criterion-related validity. Sample items are “I hope people think that I can do better than most others” and “I always avoid talking about my weakness.” Cronbach’s α were 0.87 and 0.80, respectively.

Harmony was measured by 11 items selected from Leung et al. (2011) that include the dimensions of *harmony enhancement* (6 items) and *disintegration avoidance* (5 items). Cronbach’s α were 0.86 and 0.73, respectively.

We used the 16-items scale from Triandis and Gelfand (1998) to measure vertical individualism (VI), horizontal individualism (HI), vertical collectivism (VC), and horizontal collectivism (HC), respectively. Sample items include “It is important that I do my job better than others,” “I’d rather depend on myself than others,” “It is my duty to take care of my family, even when I have to sacrifice what I want,” and “If a coworker gets a prize, I would feel proud.” Cronbach’s α were 0.80 (VI), 0.79 (HI), 0.83 (VC), and 0.76 (HC), respectively.

Six items from Dorfman and Howell (1988) and four items from Gomez-Meijia and Balkin (1989) were adopted to measure PD) and RA, respectively. The sample items are “Managers should seldom ask for the opinions of employees” and “I prefer to remain on a job that has problems that I know about rather than take the risks of working at a new job that has unknown problems even if the new job offers greater rewards.” Cronbach’s α were 0.83 (PD) and 0.81 (RA), respectively.

The translation and back-translation method was used to ensure that the statements would be well understood by respondents. All measures were 7-point Likert-type scales with poles from *strongly disagree* to *strongly agree*.

## Results

According to the two-step approach recommended by Anderson and Gerbing (1988), the measurement model was firstly examined *via* confirmatory factor analysis (CFA) with AMOS 24. The model consists of 11 constructs measured by 48 observed items. Considering the large number of constructs and items, the results indicate an overall acceptable fit: χ^2^ = 2220.2, *df* = 1035, χ^2^/*df* = 2.15, CFI = 0.84, GFI = 0.80, TLI = 0.82, RMSEA = 0.06.

In addition, the CFA results reflect that factor loadings of 46 out of the 48 items are greater than 0.5 for adequate individual item reliability (Bagozzi and Yi, 1988). Moreover, they are statistically significant (*p* < 0.05), providing evidence of convergent validity. The composite reliabilities of all the scales are greater than 0.6, which indicate sound psychometric properties (Bagozzi and Yi, 1988; Nunnally and Bernstein, 1994). Furthermore, no correlation between any two variables exceeds the square root of their AVE, demonstrating adequate discriminant validity between each construct and any other construct (Fornell and Larcker, 1981).

Next, a composite score was computed for each construct by averaging the items pertaining to that construct. Then we run a series of regression models to explore the influence of traditional cultural concepts on each value dimension. The results in Table 1 show that the dimensions of face and harmony relates to at least two value dimensions, with the explained variance ranging from 0.09 to 0.27. Specifically, *desire to gain face* positively relates to HI (*r* = 0.34, *p* < 0.001) and VI (*r* = 0.40, *p* < 0.001) but negatively affects RA (*r* = −0.13, *p* < 0.05). *Fear of losing face* exerts positive impacts on PD belief (*r* = 0.26, *p* < 0.001) and RA (*r* = 0.40, *p* < 0.001). *Harmony enhancement* positively correlates with VC (*r* = 0.11, *p* < 0.05) and HC (*r* = 0.24, *p* < 0.001), but substantially weakens PD belief (*r* = −0.43, *p* < 0.001). *Disintegration avoidance* promotes VC (*r* = 0.39, *p* < 0.001), HC (*r* = 0.25, *p* < 0.001), VI (*r* = 0.15, *p* < 0.001), PD belief (*r* = 0.34, *p* < 0.001), and RA (*r* = 0.14, *p* < 0.05).

**TABLE 1:** Results of the regression analyses.

	VC	HI	VI	HC	PD	RA
Desire to gain face	0.01	0.34***	0.40***	0.09	−0.01	−0.13*
Fear of losing face	−0.07	−0.04	−0.07	−0.09	0.26***	0.40***
Harmony enhancement	0.11*	−0.01	0.00	0.24***	−0.43***	−0.08
Disintegration avoidance	0.39***	−0.01	0.15**	0.25***	0.34***	0.14*
*R* ^2^	0.19	0.09	0.19	0.19	0.27	0.17

N = 332. VC, vertical collectivism; HI, horizontal individualism; VI, vertical individualism; HC, horizontal collectivism; PD, power distance; RA, risk aversion. *p < 0.05, **p < 0.01, ***p < 0.001.

## Discussion

Because of its long history, regime changes, and regional and social class discrepancies, Chinese culture is very complex. Since 1840, its traditional culture has been based on a complex product of different and even contradictory value systems: Confucianism, Taoism, and Buddhism, as well as assimilated Western values. Significant reform and a policy of opening up accelerated the process, which peaked after China joined the WTO in 2001. It has long been believed that Western and Chinese traditional cultures are in conflict. The former is based on individualism and the latter on collectivism. However, the relationships found in this study between the three “emic” indigenous concepts and “etic” values framework suggest that current thinking might be overly simplistic.

It is noteworthy that *desire to gain face* positively relates to both horizontal and VI. The pursuit of face is central to Chinese people and is a fundamental principle in social and business interactions in China and other Confucian countries (Hwang, 1987; Redding, 1990). However, there are different ways of gaining face from time to time. In pre-opening up era, the most prestigious achievement was to contribute unreservedly to one’s country and organization (Sun et al., 2014b). Today the most efficient way of gaining face is by embracing Western values, lifestyles and possessions in order to achieve distinctiveness and status (Li et al., 2015; Sun et al., 2017). Thus, endorsing individualism has become a normative way to gain face. Moreover, the pursuit of face may take some risks. For instance, Wang et al. (2019) find that consumers who desire to gain face are more motivated to choose fashion products that are often unconventional. Conversely, consumers with fear of losing face may consider purchasing fashion items as a risk and are reluctant to buy them. This can explain the opposite relationships between *desire to gain face*/*fear of losing face* and RA. Lastly, the respondents high in *fear of losing face* are likely to accept power inequality. We speculate that people who admit PD tend to pursue higher social position in order to avoid losing face in interpersonal communications, especially in a hierarchical society.

Both *harmony enhancement* (genuine harmony) and *disintegration avoidance* (instrumental harmony) positively relates to vertical and HC, demonstrating an overall link between harmony and collectivism. In addition, vertical individualists tend to use harmony as a tool to attain personal benefits. Interestingly, the two types of harmony has the completely opposite relations with PD belief, further illustrating they have distinct attributes from each other. Specifically, people who truly chase harmonious interpersonal relationship are not willing to accept power inequality; whereas individuals with high PD belief tend to maintain harmony for utilitarian objectives, and they are also cautious about risk.

### Implications

The findings in this study confirm the proposition that China’s current values shift during the process of modernization is based on its own culture, history, and other socio-economic factors. For instance, today Chinese do pursue personal goals and benefits instead of sacrificing themselves to the organization or the country (Faure and Fang, 2008). However, this seemingly advanced cultural expression of western culture is essentially affected by indigenous concepts like face and harmony, demonstrating that traditional culture still plays a crucial role in regulating individuals’ behavior at the deeper level.

In China and other Eastern countries where interpersonal influence is strong, people tend to endorse an interdependent sense-of-self that focuses on communal goals, norms, and obligations (Markus and Kitayama, 1991). Observing social norms and meeting others’ expectations are important sources of life satisfaction (Suh et al., 1998). Individuals who do not conform to the majority might be marginalized or excluded (Fischer, 2006). The relatively uniform ideology in China is also strengthened by its historically centralized institutional structure. Regime changes or policy renewals lead to a swift transfer of social norms from the top down through the whole society. Thus it is not hard to understand the dramatic shift from communism to consumerism and from egalitarianism to materialism since China’s reform and opening-up policy.

Therefore, when analyzing social phenomena in China, researchers must consider the indigenous cultural context because it provides the foundation for perceiving what is meaningful, relevant, and salient (Kim et al., 2000). For instance, scholars differentiate social-oriented (Eastern-style, based on others’ or group’s expectation or respect) from individual-oriented (Western-style, based on personal inclination) when conceptualizing and measuring subjective well-being (Lu and Gilmour, 2006), self (Lu, 2008), and achievement (Yu and Yang, 1994). Accordingly, when trying to understand the rise of individualism in China and taking into account its cultural context, it could be said that individualism has become a social norm that people want to conform to. As an example, when analyzing Chinese responses to modernization, Yang (1998) notes that the main motivation for young people’s new-wave behaviors in Taiwan—such as dressing in the latest fashions, drinking imported wine, and visiting underground clubs—is not to express individualistic autonomy, but to conform to contemporary mainstream norms. We propose that individualism in China should be interpreted as “social-oriented individualism”—a style of individualism that conforms to others’ expectations and gains social approval. Thus traditional culture might not be in conflict with Western values as previously expected.

In practice, because university students will be the major workforce and consumers in the near future, it is quite important to understand the new generation’s values and mindsets. Considering the huge market and great economic growth, this study provides insights for the practitioners from other regions who plan to do business in China.

### Limitation and Future Research

The current study has some limitations. First, our sample is only from one province in China. Considering the heterogeneity of culture across China (Zhang et al., 2007; Ralston et al., 2018), in the future scholars should extend the research to other regions. Second, there are some other traditional concepts that substantially regulate Chinese people’s daily life, such as guanxi, renqing, zhongyong, and so on. Future studies should analyze their impacts on personal values.

## Data Availability Statement

The raw data supporting the conclusions of this article will be made available by the authors, without undue reservation.

## Ethics Statement

Ethical review and approval was not required for the study on human participants in accordance with the local legislation and institutional requirements. Written informed consent for participation was not required for this study in accordance with the national legislation and the institutional requirements.

## Author Contributions

JS and GS developed the theoretical framework and worked on literature review and manuscript writing. JS was in charge of data collection and analysis. RC took part in literature review and data analysis. All authors contributed to the article and approved the submitted version.

## Conflict of Interest

The authors declare that the research was conducted in the absence of any commercial or financial relationships that could be construed as a potential conflict of interest.

## Publisher’s Note

All claims expressed in this article are solely those of the authors and do not necessarily represent those of their affiliated organizations, or those of the publisher, the editors and the reviewers. Any product that may be evaluated in this article, or claim that may be made by its manufacturer, is not guaranteed or endorsed by the publisher.
